# An expanded fish-based index of biotic integrity for Great Lakes coastal wetlands

**DOI:** 10.1007/s10661-018-6950-6

**Published:** 2018-09-10

**Authors:** Matthew J. Cooper, Gary A. Lamberti, Ashley H. Moerke, Carl R. Ruetz, Douglas A. Wilcox, Valerie J. Brady, Terry N. Brown, Jan J. H. Ciborowski, Joseph P. Gathman, Greg P. Grabas, Lucinda B. Johnson, Donald G. Uzarski

**Affiliations:** 1 Mary Griggs Burke Center for Freshwater Innovation, Northland College, 1411 Ellis Ave, Ashland, WI 54806 USA; 2 Department of Biological Sciences, University of Notre Dame, Notre Dame, IN USA; 3 Aquatic Research Laboratory, Lake Superior State University, Sault Ste. Marie, MI USA; 4 Annis Water Resources Institute, Grand Valley State University, Muskegon, MI USA; 5 Department of Environmental Science and Biology, SUNY College at Brockport, Brockport, NY USA; 6 Natural Resources Research Institute, University of Minnesota Duluth, Duluth, MN USA; 7 United State Environmental Protection Agency, Mid-continent Ecology Division, Duluth, MN USA; 8 Department of Biological Sciences, University of Windsor, Windsor, ON Canada; 9 Department of Biology, University of Wisconsin-River Falls, River Falls, WI USA; 10 Canadian Wildlife Service, Environment and Climate Change Canada, Toronto, ON Canada; 11 Institute for Great Lakes Research, CMU Biological Station, and Department of Biology, Central Michigan University, Mount Pleasant, MI USA

**Keywords:** Bioassessment, Biotic indicator, Coastal marsh, Fish, Wetland, Water quality, Land use, Laurentian Great Lakes

## Abstract

Biotic indicators are useful for assessing ecosystem health because the structure of resident communities generally reflects abiotic conditions integrated over time. We used fish data collected over 5 years for 470 Great Lakes coastal wetlands to develop multi-metric indices of biotic integrity (IBI). Sampling and IBI development were stratified by vegetation type within each wetland to account for differences in physical habitat. Metrics were evaluated against numerous indices of anthropogenic disturbance derived from water quality and surrounding land-cover variables. Separate datasets were used for IBI development and testing. IBIs were composed of 10–11 metrics for each of four vegetation types (bulrush, cattail, water lily, and submersed aquatic vegetation). Scores of all IBIs correlated well with disturbance indices using the development data, and the accuracy of our IBIs was validated using the testing data. Our fish IBIs can be used to prioritize wetland protection and restoration efforts across the Great Lakes basin. The IBIs will also be useful in monitoring programs mandated by the *Agreement between Canada and the United States of America on Great Lakes Water Quality*, such as for assessing Beneficial Use Impairments (BUIs) in Great Lakes Areas of Concern, and in other ecosystem management programs in Canada and the USA.

## Introduction

Human activities that alter physical, chemical, or biological processes of aquatic ecosystems also affect the structure of resident biotic communities (Karr 1981; Fausch et al. 1984). In many ecosystems, biotic indicators measured at a single point in time provide a more representative, sensitive, and time-integrated measure of conditions than typical chemical or physical measures (Karr 1981; Marchant et al. 2006; Reavie et al. 2006). The United States Environmental Protection Agency (USEPA) and other government agencies began conducting biological assessments of aquatic ecosystems in the late 1980s (Plafkin et al. 1989; Karr 1991), and their use continues to increase as new biotic indicators are developed (Ruaro and Gubiani 2013). The Index of Biotic Integrity (IBI) approach, in particular, has emerged as an effective tool for monitoring aquatic ecosystem health (Karr 1981; Belpaire et al. 2000; Ruaro and Gubiani 2013; Simon and Evans 2017). In the three decades since the IBI and its applications (e.g., Rapid Bioassessment Protocol; Plafkin et al. 1989; Barbour et al. 1999) were first introduced, biotic indicators have been developed for numerous taxonomic groups inhabiting a wide variety of aquatic ecosystems, including coastal wetlands of the Laurentian Great Lakes (e.g., Burton et al. 1999; Seilheimer and Chow-Fraser 2006; Grabas et al. 2012).

Coastal wetlands occur throughout the Great Lakes where hydrology (e.g., wave and current energy) is sufficiently quiescent for emergent vegetation to persist, and where sediment is conducive to macrophyte growth (Albert et al. 2005). These shallow, productive ecosystems provide critical habitat for many fish species of ecological and economic importance (Chubb and Liston 1986; Klarer and Millie 1992; Parker et al. 2012). Unfortunately, half of the coastal wetland area that was present before European settlement has been converted to other land uses (Maynard and Wilcox 1997), and many remaining wetlands are impacted by invasive species, fragmentation, nutrient loading, and hydrologic manipulation (Bedford 1992; Wilcox 1995; SOLEC 2007; Cooper et al. 2012). Therefore, coastal wetland restoration and protection are vital components of long-term management of the Great Lakes (Sierszen et al. 2012). Broad-scale monitoring is essential for identifying wetlands most in need of protection or restoration, to evaluate the effectiveness of restoration projects, and for both local and regional time-trend analysis.

Government agencies in both Canada and the USA initiated a process to develop indicators of ecosystem health for coastal wetlands and other Great Lakes habitats at the State-of-the-Lakes Ecosystem Conferences (SOLEC) in 1998 and 2000. Recommended indicators included IBIs based on fishes, invertebrates, and plants, even though no broadly accepted protocols were available at the time for any of these groups. Subsequent efforts by members of the Great Lakes Coastal Wetlands Consortium (GLCWC), the Great Lakes Environmental Indicators group (GLEI; Niemi et al. 2007), and others developed a variety of biotic indicators. Fish-based indicators are particularly valuable because fish are sensitive to many types of human disturbance; they occupy multiple trophic levels, and the economic and esthetic benefit of protecting fish communities is greater and more visible than for many other groups (Harris 1995; Oberdorff et al. 2001).

After marked progress in indicator development during the 2000s, the USEPA initiated a Great Lakes basin-wide coastal wetland monitoring program based on GLCWC protocols in 2011 (Uzarski et al. 2017). The monitoring program includes sampling of fish, macroinvertebrate, bird, amphibian, and vegetation communities, as well as water quality. Protocols for fish monitoring call for sampling fish within discrete mono-dominant “vegetation zones” at each wetland so that IBI scores could be calculated using a habitat-stratified approach (Uzarski et al. 2005, 2017). However, when the monitoring program began in 2011, fish-based IBIs had been developed only for bulrush (*Schoenoplectus* spp.) and cattail (*Typha* spp.) vegetation zones (Uzarski et al. 2005), which represent only a portion of the habitat types encountered in Great Lakes coastal wetlands. Furthermore, these previously developed IBIs were generated using a much smaller dataset than what is currently available. We built on the approach of Uzarski et al. (2005) and formulated fish-based IBIs for the most common vegetation types encountered in Great Lakes coastal wetlands using data collected during the first 5 years (2011–2015) of the Great Lakes Coastal Wetland Monitoring Program (Uzarski et al. 2017). Our objectives in the current paper are to describe our approach to developing the expanded fish-based IBIs and to test these IBIs against anthropogenic disturbance gradients derived from water quality and surrounding land-use data for a 5-year period of wetland monitoring. We predicted that these refined fish IBIs would accurately reflect the degree of human disturbance in Great Lakes wetlands.

## Methods

### Study area and site selection

Fish communities and water quality variables were sampled from 2011 to 2015 at 470 coastal wetlands located throughout all five Laurentian Great Lakes and connecting channels. Sites were selected according to a probabilistic randomized design developed previously by the GLCWC (Uzarski and Otieno 2008; Uzarski et al. 2017). All coastal wetlands greater than 4 ha in area with herbaceous vegetation and a surface water connection to a Great Lake or connecting channel were eligible for sampling. Wetlands associated with tributaries were included if their hydrology was influenced by Great Lakes water levels and they were located within 1 km of a shoreline of a Great Lake or connecting channel. An exception was drowned river mouth wetlands (Albert et al. 2005) along the eastern shore of Lake Michigan, which often were located farther than 1 km upstream from the coast but were included because hydrology in these wetlands is dictated by Lake Michigan water levels. The full population of potentially sampleable wetlands was identified using a spatial database compiled previously by Albert and Simonson (2004) and Ingram and Potter (2004), who also classified the geomorphic type of each wetland (i.e., riverine, barrier-protected, or lacustrine). A three-way stratified sampling approach was used: the 3 strata were Great Lake (*N* = 5), wetland type (*N* = 3), and ecoregion (*N* = 3, Bailey and Cushwa 1981; Omernik 1987). Each wetland was randomly assigned to 1 of the 5 sampling years, but adherence to the stratification constraints ensured that all strata were proportionally represented in each sampling year. The monitoring program was designed to assess wetlands over a 5-year cycle such that 20% of wetlands in each stratum were assigned to each sampling year (Uzarski et al. 2017).

Wetlands were not sampled if field crews could not safely reach the site, access through private property was denied, a wetland no longer existed at the location, a surface-water connection to the Great Lake or connecting channel could not be identified (e.g., diked wetlands), only woody vegetation was found at the site, or if water depth was not appropriate for fish sampling. Fish communities and water quality were sampled from mid-June to early September each year, starting in the southern portion of the basin in June and working northward as the summer progressed so that sampling coincided with maximum vegetation biomass.

### Fish sampling

Fish sampling was conducted within discrete vegetation zones at each wetland (Uzarski et al. 2017). Vegetation zones were defined as patches of vegetation in which a particular macrophyte genus (e.g., *Schoenoplectus*, *Typha*) or growth form (e.g., “lily,” referring to floating-leaved genera, such as *Brasenia* spp.*, Nuphar* spp.*, Nymphaea* spp.) dominated the plant community based on visually assessed coverage estimates. Other macrophyte genera or growth forms often occurred within a given vegetation zone; however, zones were comprised of at least 75% of the given genus or growth form. The vegetation zone-specific sampling approach was adopted by the GLCWC to account for habitat variability among wetlands with different vegetation types and to ensure that our IBIs could be used over a range of Great Lakes water levels (Burton et al. 1999; Uzarski et al. 2004). Great Lakes water levels can fluctuate dramatically among years, which may create problems because vegetation zones extend downslope during prolonged (e.g., 2–3 years or more) low-water periods and upslope when water levels are high (Wilcox et al. 2002; Gathman et al. 2005). Thus, stratification of data collection by vegetation zone allows the IBIs to be used at various water levels (Uzarski et al. 2005, 2017).

At each wetland, we sampled all available vegetation zones with water depths between 20 and 100 cm (the minimum and maximum depths at which our gear functioned efficiently) and large enough for three fyke nets to be placed at least 25 m apart to avoid net interference. In wetlands where multiple disjunct smaller patches of the same vegetation type were present, these smaller patches were sampled by placing single nets in different patches, as long as they met our minimum-size criterion of 100 m^2^. For each vegetation zone, three replicate fyke nets were set overnight (usually 16–18 h). Fyke nets were set individually (not paired). Two net sizes were used, depending on water depth at the net location. Small nets (0.9-m wide × 0.5-m tall boxes) were set in water 20–45 cm deep, and large nets (1.2-m wide × 1.0-m tall boxes) were set in water 45–100 cm deep. Data from large and small nets were treated in the same way because the main difference between large and small nets was the box height, which was at or above the water surface for all sets. Leads (7.3 m long) were attached to the middle of net boxes and were extended straight out from the box. Wings (1.8 m long) were set at 45° angles to the lead. All mesh was of 0.5 cm bar measurement. Net boxes, wings, and leads were usually set completely within the vegetation zone of interest and oriented so that leads point toward shore. In very dense cattail stands, net boxes were set just outside of the vegetation with wings and leads extending into the vegetation itself, perpendicular to the leading edge of the vegetation. Fish were identified to species, measured, and counted in the field. Fish less than 20 mm total length were not counted because the gear was inefficient at capturing fish that small. Difficult-to-identify specimens (e.g., small cyprinids) were returned to the laboratory for identification under a dissecting microscope.

We developed IBIs for bulrush (*Schoenoplectus* spp.), cattail (*Typha* spp.), lily (*Brasenia* spp.*, Nuphar* spp.*, Nymphaea* spp.), and submersed aquatic vegetation (SAV; primarily *Myriophyllum* spp., *Ceratophyllum* spp., *Chara* spp., and *Najas* spp.). Of all the wetlands sampled for fish by the Great Lakes Coastal Wetland Monitoring Program from 2011 to 2015, 89% had at least one of these vegetation types. Additional vegetation types (e.g., *Phragmites australis*, *Peltandra virginica, Pontederia* spp*.*, *Sparganium* spp*.*, *Juncus* spp*.,* and *Zizania* spp*.*) were occasionally sampled but at much lower frequency, and we did not attempt to develop IBIs for these zones due to small sample sizes.

### Water quality

Water quality data were used for both IBI development and testing but were not used in the final IBI metric calculations. Dissolved oxygen (% saturation), pH, and specific conductance (μS/cm) were measured at every net location using a hand-held meter such as a YSI 6600 multi-parameter sonde (Yellow Springs Instruments, Inc., Yellow Springs, OH) or similar instrument. Measurements were taken at mid water-column depth, and values from the three net locations were averaged for the vegetation zone. Water samples (l L) were collected at each net location from the middle of the water column and composited into a single sample for each vegetation zone. Clarity of the composite water sample was assessed using a 100-cm turbidity tube (Myre and Shaw 2006). The composite sample was returned to the lab and analyzed for soluble reactive P (SRP), total P, nitrate-N, ammonium-N, total N, and chlorophyll *a* using standard preservation and analytical methods (USGS 2003; APHA 2005). Additional details on water quality sampling protocols are provided in Uzarski et al. (2017).

### Land-cover

Similarly to water quality, surrounding land-cover data were used for both IBI development and testing but not in the final IBI metrics. Surrounding land-cover was quantified using the 2006 National Land-cover Database for the U.S. shoreline and the 2005 Land Information Ontario Database for Canadian watersheds. We used coastal wetland data layers generated previously by Albert and Simonson (2004) and Ingram and Potter (2004) to define the area of each of our 470 wetlands and then quantified four broad land-cover classes in 1-km and 20-km buffers surrounding each wetland. The larger buffer represented more general regional land-cover, and the smaller buffer represented land-cover in the immediate coastal margin, both of which can affect wetland water quality (Uzarski et al. 2005). Land-cover classes included agriculture, developed land, forested land, and wetland, which we expressed as percentages of total upland area within each buffer.

### Anthropogenic disturbance gradients

We used water quality and land-cover data as surrogates of anthropogenic disturbance for IBI development and subsequent testing. We chose this approach over simply measuring deviation of metric values from those of reference sites (i.e., the “reference condition approach”; Bailey et al. 2004) because true reference conditions and reference sites are difficult to identify, and because multiple types of human stressors simultaneously affect biological communities in Great Lakes coastal wetlands (Wilcox et al. 2002; Brazner et al. 2007). Incorporating various dimensions of stress (i.e., water quality and surrounding land-cover) should, therefore, provide a more comprehensive picture of human disturbance and help us to identify, calibrate, and test a robust set of biotic indicators. The surrogates of anthropogenic disturbance used in IBI development and testing included individual water quality and land-cover variables, as well as multivariate disturbance indices. Individual variables included surrounding land-cover, dissolved and total N and P concentrations, specific conductance, and turbidity. Multivariate disturbance indices were calculated by combining the above variables into principal components using principal components analysis (PCA) and by summing a set of rank-transformed variables into a composite index that we refer to as “SumRank” (Uzarski et al. 2005, 2017). Rank-transformed variables used in SumRank included turbidity, chlorophyll *a*, total P, SRP, total N, ammonium-N, nitrate-N, dissolved oxygen, pH, specific conductance, the four land-cover classes, and scores from the first principal component of the PCA calculated on the above variables. Higher values of SumRank indicate higher water quality and less-developed surrounding land (Uzarski et al. 2005, 2017). Because principal components analysis and SumRank are relative indices that vary based on the dataset used, we calculated these separately for each of the four vegetation zone types for IBI development and testing. We also used a previously published landscape-based stress index, SumRel (Host et al. 2011), as another measure of potential anthropogenic disturbance for IBI development.

### IBI development

Indices of biotic integrity were developed for each of the four vegetation types independently of one another. For each vegetation type, data were split into “development” and “testing” datasets, with 50% of wetlands randomly assigned to each subset for each vegetation type. The development dataset was used to identify fish metrics that correlated with anthropogenic disturbance gradients. The testing dataset was used to test how well the new IBIs correlated with anthropogenic disturbance indices.

### Metric identification and scoring

Fish data were summarized as catch of each species per net per night in each vegetation zone at each wetland, which we considered the catch per unit effort (CPUE) for that zone. Metric identification and IBI development generally followed previously established methods (e.g., Karr 1981; USEPA 2002; Lyons 2012) in which (1) a large set of candidate metrics was calculated; (2) metrics were tested for presence of a relationship with anthropogenic disturbance or habitat quality; (3) metrics were screened for adequate range of responses and for highly redundant metrics; (4) scoring schemes were devised for each of the final metrics; and (5) the final IBI was tested against an independent dataset (i.e., our test dataset).

The initial set of candidate metrics included those from literature sources (Karr 1981; Minns et al. 1994; Wilcox et al. 2002; Uzarski et al. 2005; Lyons 2012; Rabaru and Masese 2012) and metrics representing catches of the 25 species most frequently collected in each vegetation type. The full set of candidate metrics was calculated and evaluated for each of the four vegetation types. Candidate metrics fell into seven broad categories: (1) diversity-based (alpha diversity, Shannon diversity, evenness); (2) native vs. nonnative species occurrence and abundance; (3) community taxonomic composition; (4) feeding ecology; (5) habitat affinity; (6) species attributes (e.g., longevity and relative body size); and (7) species sensitivity or tolerance to human disturbance (see Appendix Table 3 for the full set of candidate metrics and Appendix Table 4 for species attributes). For most metrics, multiple alternative formulations were calculated such as alpha richness vs. % richness or CPUE vs. relative abundance of a species.

Our goal was to use the 2011–2015 development dataset to generate a set of IBI metrics for each vegetation type that included 10–15 metrics that each correlated with multiple surrogates of anthropogenic disturbance (i.e., water quality and land-use variables, SumRank, SumRel, principal components). We used a number of techniques to identify metrics that varied as a function of human disturbance. First, Spearman correlations were calculated between all candidate fish metrics and all disturbance indices. High correlation coefficients (Spearman’s rho > 0.5) suggested a potential relationship between the metric and the disturbance variable, and we retained those metrics for further evaluation. Second, we used SumRank scores to identify high-quality wetlands (20% of wetlands with the highest SumRank scores) and impacted wetlands (20% of wetlands with the lowest SumRank scores). Mann-Whitney *U* tests were then conducted to compare values between these two groups of wetlands for each candidate fish metric; significant differences suggested an association between the metric and human disturbance. Third, we simply plotted each fish metric against each multivariate disturbance index to evaluate graphically whether a potential relationship existed. These three techniques were used in conjunction to evaluate each candidate metric and reduce the initial set of candidate metrics to a subset for each vegetation type using the development dataset.

Given the extremely large number of comparisons made and relationships evaluated, we acknowledge the risk of retaining metrics that had spurious relationships with anthropogenic disturbance variables. To mitigate this risk, we retained only those metrics that exhibited consistent patterns with respect to multiple disturbance variables. Scores of most of the retained metrics were related to at least one water quality variable, at least one surrounding land-cover variable, and at least one of the multivariate disturbance indices (e.g., principal component scores, SumRank, and/or SumRel). Finally, because multiple alternative formulations were calculated for many of the metrics, we chose the formulation that correlated most highly to human disturbance and was robust to catches of taxa not targeted in the metric. For example, if both CPUE and relative abundance of a particular fish species correlated with anthropogenic disturbance indices, we retained the CPUE formulation because it is less sensitive to catches of other taxa in the same habitat. We then screened each of the retained metrics to determine whether anomalous catches of individual species (e.g., large schools of minnows or juvenile bullhead) were responsible for relationships with anthropogenic disturbance variables. When this occurred, the metric was removed from the candidate list. Remaining candidate metrics were then screened to ensure that they represented a sufficiently wide range in values. We eliminated richness metrics with a range of four or fewer taxa, percent richness metrics with a range of less than 25%, CPUE metrics with a range of fewer than five fish, and relative abundance metrics with a range of less than 20%. Remaining candidate metrics were then evaluated for redundancy using correlation analyses (Spearman’s rho > 0.8). While we expected to observe some correlation among the selected metrics given they all were chosen to reflect anthropogenic disturbance, we removed highly redundant metrics, especially metrics that represented similar aspects of fish community composition. For example, *% Carnivore* (invertivore+piscivore+zooplanktivore) *richness* and *% Piscivore richness* were highly correlated in both the water lily and SAV zones and represent similar aspects of fish community structure; therefore, *% Carnivore richness* was retained in both of these IBIs.

Next, scoring schemes were devised for each metric by graphically analyzing scatter plots of fish metric values against the disturbance indices. In most cases, natural breaks observed in the data were used to establish scoring thresholds, and a three-category scoring scheme was devised for each metric, corresponding to metric scores of 0, 1, or 2, with higher scores implying greater biotic integrity. These steps resulted in a set of IBI metrics, along with scoring thresholds for each of the four vegetation types. Summing of the metric scores for each vegetation zone sampled yielded vegetation zone-level IBI scores.

A final optimization procedure was conducted in which metrics that did not improve the overall correlation between IBI scores and SumRank were identified, again using only the development dataset. This step was accomplished by re-calculating IBI scores and SumRank–IBI correlations (Spearman’s rho) after sequentially leaving out each metric. Metrics that did not improve the fit of the IBI to the disturbance index were omitted. This step resulted in a final set of 10 metrics for the cattail and water lily IBIs, and 11 metrics for bulrush and SAV IBIs. Scores were then summed across the 10–11 metrics for each vegetation zone sampled, divided by the total number of points possible for that zone (either 20 or 22), and then multiplied by 100 to rescale the zone-level IBI scores to a 0–100 point range. Therefore, IBI scores closer to zero implied a greater degree of human impact and low habitat quality, whereas IBI scores closer to 100 implied a low degree of human impact and high habitat quality.

### IBI testing

Using the IBI metrics, scoring thresholds, and re-scaling formulae derived with the development dataset, IBI scores for each of the four vegetation types were calculated using the test dataset. These IBI scores were then compared to SumRank scores using Pearson correlation coefficients. We used the SumRank index to test the IBIs because it integrates both water quality and surrounding land-cover so we considered it the most robust index of anthropogenic disturbance available. Inter-annual variability in IBI performance was evaluated in two ways. First, we calculated SumRank–IBI correlations for each individual year (2011–2015) using the full dataset (development and testing data combined) to determine whether the IBIs were consistently related to anthropogenic disturbance throughout the 5-year period. Second, we used analyses of covariance (ANCOVA, homogenous slopes model) to test the effects of year while accounting for the relationship between IBI scores and SumRank.

### Biotic integrity categories

After developing and testing the IBIs for each vegetation type (i.e., different sets of metrics for each of the four vegetation types), we calculated mean IBI scores at the wetland level by averaging the zone-level scores for each wetland (0–100 point scale). We then assigned individual wetlands to biotic integrity categories as in Karr et al. (1986), Uzarski et al. (2004), and Lyons (2012). Category class names were “reference quality,” “mildly impacted,” “moderately impacted,” “moderately degraded,” and “degraded.” Category class boundaries were determined by assigning wetlands to approximate quintile bins based on final wetland-level mean IBI scores.

## Results

### Fish collections

A total of 113 species and over 359,000 fishes were collected over the 5 years of the study. Mean (±SE) species richness was similar among lakes, ranging from 9.5 ± 0.2 species per vegetation zone in Lake Ontario (*n* = 176 individual vegetation zones) to 10.9 ± 0.5 in Lake Superior (*n* = 78). Mean CPUE (expressed as individuals net-night^−1^, all species combined) was greatest in Lake Michigan (407 ± 159, *n* = 100 vegetation zones), in part due to one outlier (15,836 fish net^−1^ night^−1^). After removing this outlier, mean CPUE remained greater in Lake Michigan (257 ± 52) than in lakes Superior (203 ± 40, *n* = 78), Erie (177 ± 44, *n* = 81), Huron (150 ± 19, *n* = 217), and Ontario (72 ± 9, *n* = 176).

### IBI metric selection and testing

Metric selection procedures conducted with the development data yielded 10–11 metrics for each vegetation type. Most of these metrics had values that correlated with multiple disturbance variables, including the multivariate disturbance indices (Table 1). A number of metrics functioned well in several of the IBIs (Table 1); metrics ultimately used in all four IBIs included *Nonnative species richness* and *% Richness of species particularly sensitive to environmental degradation* [sensitivity based on Plafkin et al. 1989 and Lyons 2006, 2012]. Metrics selected for three of the four IBIs included *Rock bass CPUE*, *Black+brown bullhead relative abundance*, and *Evenness*. While our goal was to identify at least one metric from each of the seven categories of integrity for each IBI, we could not identify a habitat-affinity metric for either the bulrush or SAV IBI, a diversity or feeding ecology metric for the cattail IBI, nor a species attribute (e.g., longevity and relative body size) metric for the lily IBI (Table 1, Appendix Table 3).

**Table 1:** Spearman’s correlation coefficients (r_s_) between index of biotic integrity (IBI) metrics and multivariate disturbance indices (SumRank, principal component 1 [PC1], SumRel [Host et al. 2011]) for the four vegetation types sampled in the development dataset only

	SumRank	PC1	SumRel
Bulrush (*Schoenoplectus* spp.; *n* = 96)			
Evenness	0.041	− 0.072	0.016
Nonnative species richness	− 0.097	0.161	0.051
Native Cyprinidae CPUE	0.131	− 0.113	− 0.159
Smallmouth bass CPUE	0.243*	− 0.121	− 0.216*
% Black+brown bullhead	0.258*	− 0.197	− 0.172
Johnny darter CPUE	0.286**	− 0.455***	− 0.287*
Common carp CPUE	− 0.283**	0.227*	0.250*
% Carnivore (invertivore+piscivore+zooplanktivore)	− 0.068	0.080	− 0.034
% Richness of high and extra-high temperature spawners	− 0.240	0.285**	0.413**
% Richness short-lived species	0.129	− 0.073	− 0.234*
% Richness species particularly sensitive to environmental degradation	0.297**	− 0.259*	− 0.136
Cattail (*Typha* spp.; *n* = 72)			
% Richness native species	0.609***	− 0.554***	− 0.370**
Nonnative species richness	− 0.575***	0.520***	0.356**
% Native Cyprinidae	− 0.177	0.076	− 0.082
Rock bass CPUE	0.212*	− 0.247*	− 0.075
% Black+brown bullhead	0.274*	− 0.307**	− 0.407**
% Richness benthic habitat species	− 0.417***	0.350**	0.125
% Richness nest spawners	0.498***	− 0.394**	− 0.226*
% Richness of high and extra-high temperature spawners	− 0.134	0.302*	0.088
% Richness large and extra large species	− 0.243*	0.226*	0.078
% Richness species particularly sensitive to environmental degradation	0.073	− 0.093	− 0.041
Water lily (*Brasenia* spp., *Nuphar* spp., *Nymphaea* spp.; *n* = 76)			
Evenness	0.341***	− 0.346**	− 0.435***
Nonnative species richness	− 0.559***	0.524***	0.388***
Rock bass CPUE	0.527***	− 0.500***	− 0.281**
Smallmouth bass CPUE	0.412***	− 0.400***	− 0.110
% Black+brown bullhead	0.397***	− 0.383***	− 0.184*
Yellow perch CPUE	0.226*	− 0.183*	− 0.165
% Common carp	− 0.439***	0.419***	0.367***
% Richness carnivore species (invertivore+piscivore+zooplanktivore)	0.432***	− 0.371***	− 0.378***
% Richness vegetation spawners	0.131	− 0.164*	0.144
% Richness species particularly sensitive to environmental degradation	0.228	− 0.258*	− 0.211*
Submersed aquatic vegetation (*n* = 87)			
Evenness	0.259*	− 0.145	− 0.034
Nonnative species richness	− 0.242*	0.221*	0.158
% Richness native species	0.315**	− 0.339**	− 0.241*
% Native Cyprinidae	0.189*	− 0.255*	− 0.099
Johnny darter CPUE	0.268*	− 0.325**	− 0.102
Rock bass CPUE	0.213*	− 0.183*	− 0.098
% Common carp	− 0.309**	0.276**	0.177
% Richness carnivore species (invertivore+piscivore+zooplanktivore)	0.160	− 0.145	− 0.091
% Richness large and extra large species	− 0.276**	0.212*	0.193*
% Richness short-lived species	0.117	− 0.096	− 0.166
% Richness species particularly sensitive to environmental degradation	0.240*	− 0.250*	− 0.345**

*p < 0.1; **p < 0.01; ***p < 0.001

**Fig. 1:**
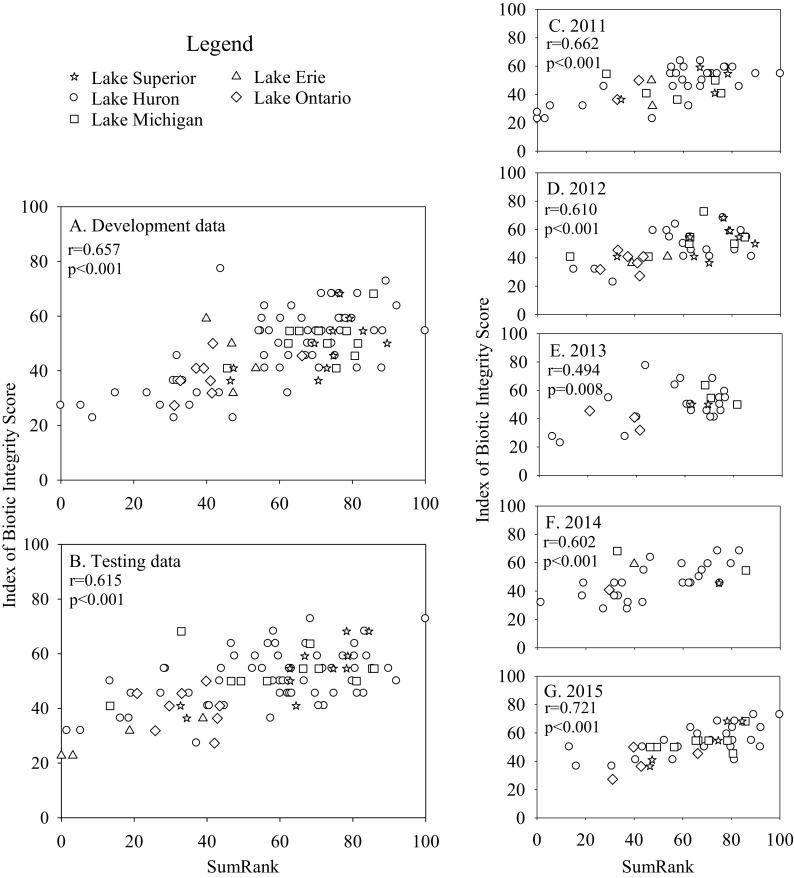
Relationships between bulrush zone IBI scores and SumRank indices calculated for the development (**a**) and test datasets (**b**), as well as for individual years (**c–g**). Development and test data were combined for the individual year analyses. Separate SumRank values were calculated for each comparison. Pearson correlations were calculated between IBI and SumRank scores

**Fig. 2:**
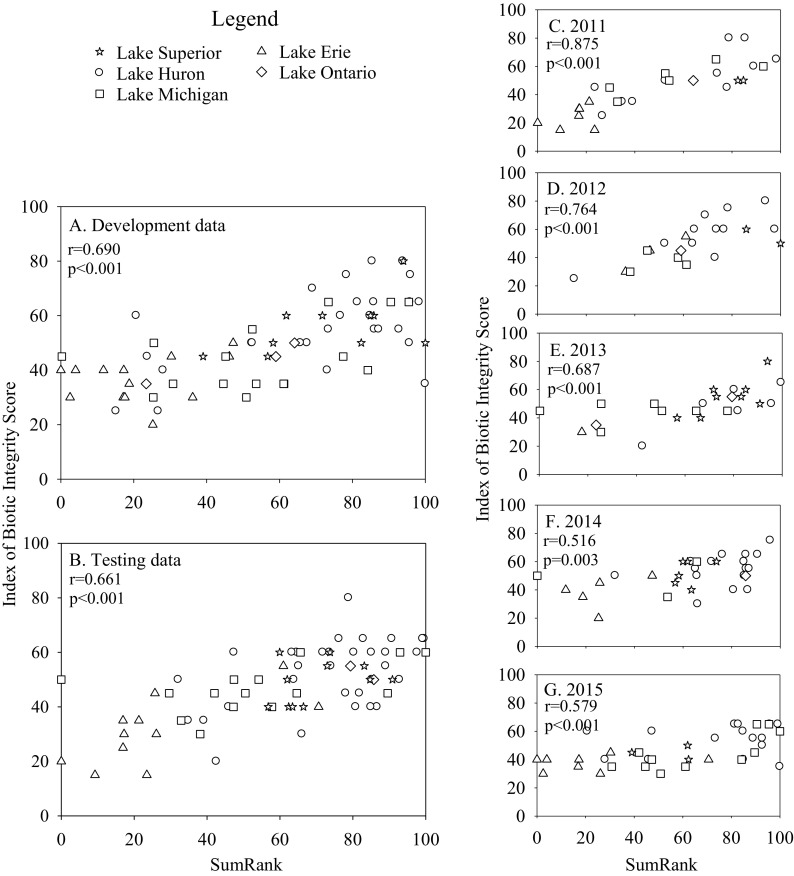
Relationships between cattail zone IBI scores and SumRank indices calculated for the development (**a**) and test datasets (**b**), as well as for individual years (**c–g**). Development and test data were combined for the individual year analyses. Separate SumRank values were calculated for each comparison. Pearson correlations were calculated between IBI and SumRank scores

**Fig. 3:**
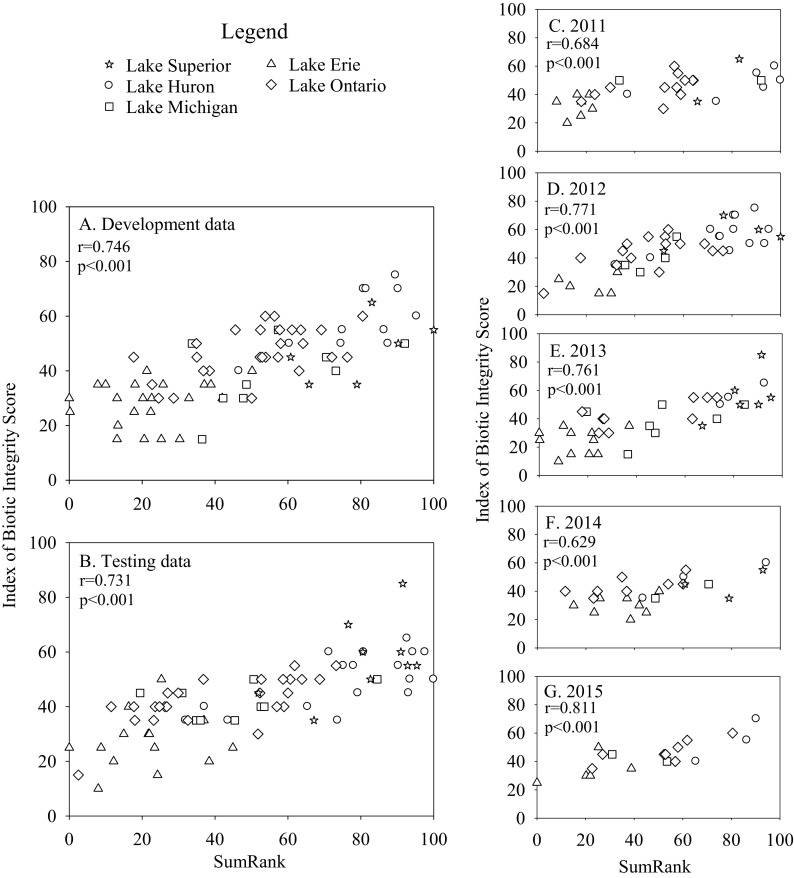
Relationships between water lily zone IBI scores and SumRank indices calculated for the development (**a**) and test datasets (**b**), as well as for individual years (**c–g**). Development and test data were combined for the individual year analyses. Separate SumRank values were calculated for each comparison. Pearson correlations were calculated between IBI and SumRank scores

**Fig. 4:**
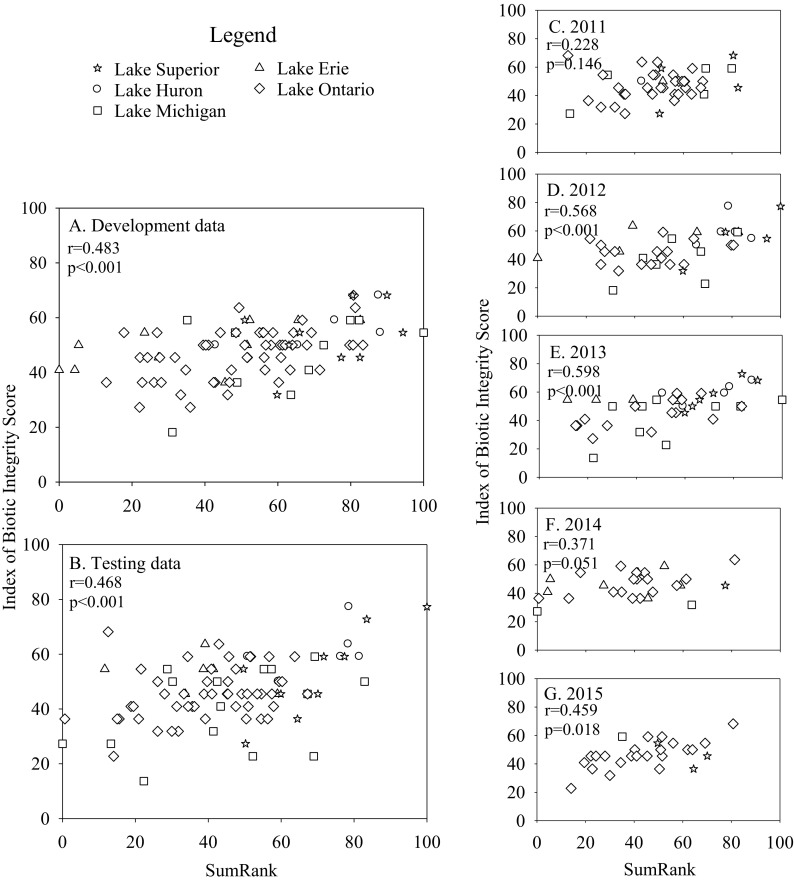
Relationships between submersed aquatic vegetation zone IBI scores and SumRank indices calculated for the development (**a**) and test datasets (**b**), as well as for individual years (**c–g**). Development and test data were combined for the individual year analyses. Separate SumRank values were calculated for each comparison. Pearson correlations were calculated between IBI and SumRank scores

Scoring thresholds were established for each of the selected metrics in each of the four IBIs (Table 2). After scoring thresholds were established, scores for each metric were determined for each vegetation zone in the development data set. For example, if a bulrush zone had an average evenness value of 0.6, then that zone would receive a score of 1 for that metric (Table 2); if that same zone had nonnative species richness of 0, then it would receive a score of 2 for that metric (Table 2). This process was completed for each metric for each zone sampled—bulrush metrics were applied to the fish data collected in bulrush zones, cattail metrics were applied to data collected in cattail zones, and so on. Metric scores (0, 1, or 2) were then summed across the set of metrics for each vegetation zone and converted to a percentage of total points possible for that zone type (22 points for bulrush and SAV; 20 points for cattail and lily [Table 2]). This yielded zone-level IBI scores for each of the four vegetation types. The IBI scores for bulrush, cattail, and lily correlated well (*r* ≥ 0.657, *p* < 0.001) with SumRank using the development dataset (Figs. 1, 2, and 3). The relationship between IBI scores and SumRank was less strong for SAV but still statistically significant (*r* = 0.483, *p* < 0.001; Fig. 4). When the IBIs were tested using the test dataset, IBIs for bulrush, cattail, and lily remained strongly correlated (*r* ≥ 0.615, *p* < 0.001) with SumRank at the basin-scale (Figs. 1, 2 and 3). For SAV, the correlation between IBI scores and SumRank with the test dataset was again lower than for the other vegetation types but remained statistically significant (*r* = 0.468, *p* < 0.001; Fig. 4).

**Table 2:** Final IBI metrics and scoring thresholds for the four vegetation zone types. Catch per unit effort (CPUE) was catch net^−1^ night^−1^. Final zone scores are calculated by re-scaling the sum of all metrics for a vegetation zone to a 100-point scale. Appendix Table 4 contains taxa trait information

	Scoring		
	0	1	2
Bulrush (*Schoenoplectus* spp.)			
Evenness	0–0.4	> 0.4–0.8	> 0.8
Nonnative species richness	≥ 2	1	0
Native Cyprinidae CPUE	0	> 0–50	> 50
Smallmouth bass CPUE	< 2	2–5	> 5
% Black+brown bullhead	0	> 0–25	> 25
Johnny darter CPUE	0	> 0–0.34	> 0.34
Common carp CPUE	> 2	> 0–2	0
% Carnivore (invertivore+piscivore+zooplanktivore)	> 90	40–90	< 40
% Richness of high and extra-high temperature spawners	100	> 82–100	0–82
% Richness short-lived species	< 20	20–60	> 60
% Richness species particularly sensitive to environmental degradation	0	> 0–15	> 15
Final score for zone = (sum of metrics / 22) * 100			
Cattail (*Typha* spp.)			
% Richness native species	< 60	60–< 100	100
Nonnative species richness	> 2	1–2	0
% Native Cyprinidae	0–20	> 20–50	> 50
Rock bass CPUE	0	> 0–3	> 3
% Black+brown bullhead	0	> 0–25	> 25
% Richness benthic habitat species	> 75	30–75	< 30
% Richness nest spawners	0	> 0–70	> 70
% Richness of high and extra-high temperature spawners	100	60–< 100	< 60
% Richness large and extra-large species	> 40	20–40	< 20
% Richness species particularly sensitive to environmental degradation	0	> 0–8	> 8
Final score for zone = (sum of metrics / 20) * 100			
Water lily (*Nuphar advena* sp., *Nymphaea odorata* sp.)			
Evenness	< 0.5	0.5–0.75	> 0.75
Nonnative species richness	> 2	> 0–2	0
Rock bass CPUE	< 2	2–6	> 6
Smallmouth bass CPUE	0	> 0–3	> 3
% Black+brown bullhead	< 5	5–30	> 30
Yellow perch CPUE	0	> 0–10	> 10
% Common carp	> 3	> 0–3	0
% Richness carnivore species (invertivore+piscivore+zooplanktivore)	< 50	50–75	> 75
% Richness vegetation spawners	< 15	15–40	> 40
% Richness species particularly sensitive to environmental degradation	0	> 0–10	> 10
Final score for zone = (sum of metrics / 20) * 100			
Submersed aquatic vegetation			
Evenness	< 0.2	0.2–0.80	> 0.80
Nonnative species richness	> 3	1–3	0
% Richness native species	< 75	75–95	> 95
% Native Cyprinidae	< 20	20–60	> 60
Johnny darter CPUE	0	> 0–2	> 2
Rock bass CPUE	0	> 0–5	> 5
% Common carp	> 5	> 0–5	0
% Richness carnivore species (invertivore+piscivore+zooplanktivore)	< 50	50–80	> 80
% Richness large and extra-large species	> 40	> 8–40	0–8
% Richness short-lived species	< 20	20–70	> 70
% Richness species particularly sensitive to environmental degradation	< 5	5–20	> 20
Final score for zone = (sum of metrics / 22) * 100

Evaluating the IBIs for individual years (development and test datasets combined) suggested that the IBIs for bulrush, lily, and cattail were robust to inter-annual variation. Relationships between IBI scores and SumRank were strong for each individual year (Figs. 1, 2, and 3), despite the smaller number of observations used in each correlation. The IBI for SAV correlated significantly with SumRank in 2012, 2013, and 2015, but not in 2011 or 2014 (Fig. 4). Analyses of covariance revealed that sampling year did not have a significant effect on IBI scores for any of the vegetation types (*p* > 0.3).

### Biotic integrity categories

**Fig. 5:**
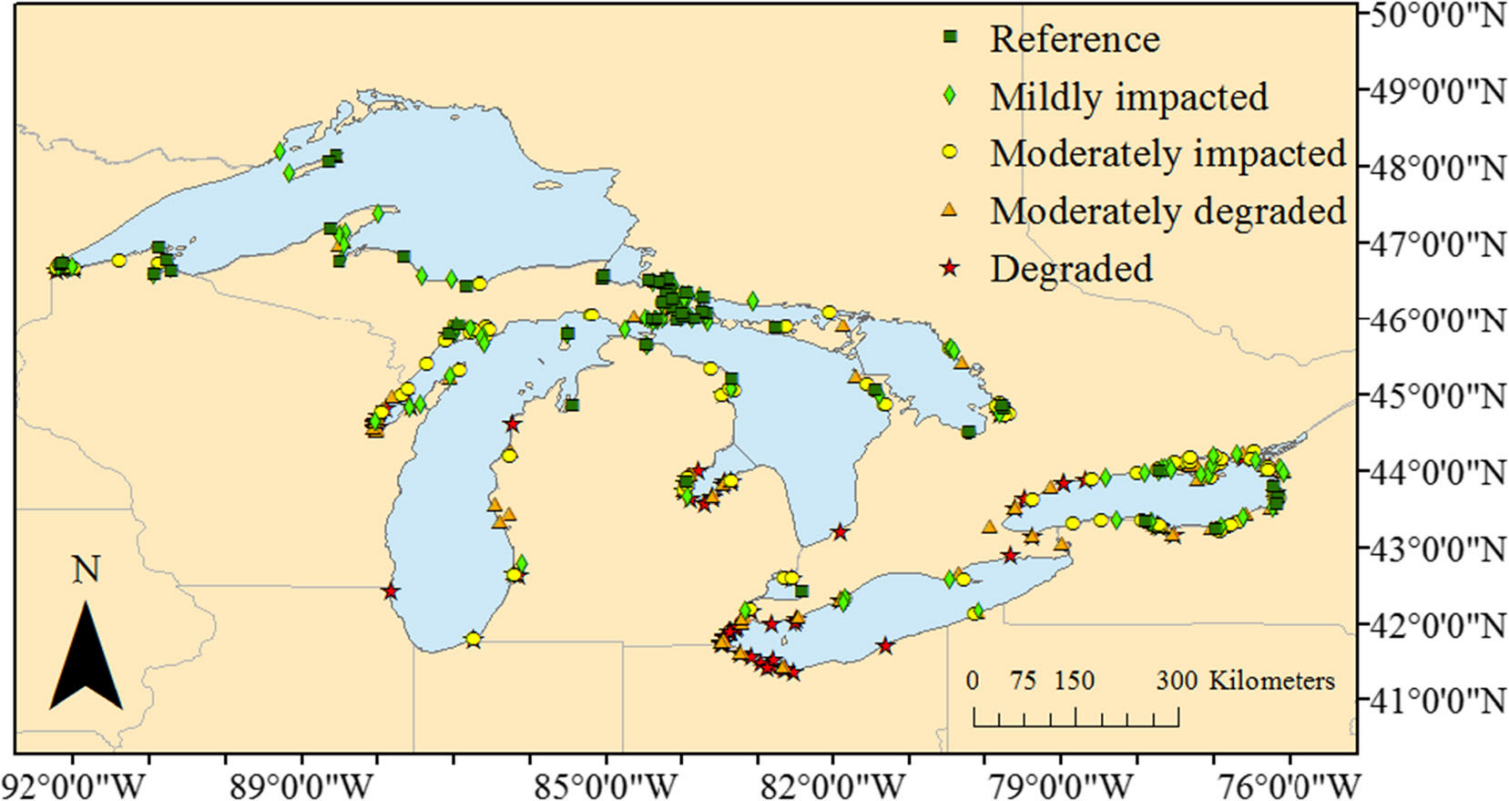
Biotic integrity categories based on fish IBI scores (means of IBI scores from all vegetation zones sampled per wetland, both development and testing data sets). Categories correspond to approximate quintiles of the full set of IBI scores for the basin. The downstream boundary for the region was a line through Wolfe Island in the St. Lawrence River, from Kingston, Ontario to Cape Vincent, New York

We used mean IBI scores (average of the 1–4 vegetation zones sampled within each wetland) to assign wetlands to categories, each representing approximately 20% of all wetlands sampled (Fig. 5). Category ranges were < 36 = degraded; 36 to 45 = moderately degraded; > 45 to 50 = moderately impacted; > 50 to 60 = mildly impacted; > 60 = reference quality. Lake Erie had the highest percentage of its wetlands in the degraded category (56%) whereas Lake Superior had the lowest percentage of its wetlands in the degraded category (6%). Lakes Superior and Huron had the highest percentage of wetlands in the reference category (27 and 22%, respectively), whereas lakes Erie and Ontario had the lowest percentages of wetlands in the reference category (2% [1 wetland] and 5% [6 wetlands], respectively).

## Discussion

We developed fish assemblage-based IBIs for the four most common vegetation types encountered in Great Lakes coastal wetlands using the most extensive dataset collected to date for these wetlands. Because at least one of these four vegetation types occurred in 89% of wetlands sampled for fish basin-wide, our IBIs have the potential to enhance the utility of existing and future monitoring programs greatly. The IBI scores correlated well with an index of anthropogenic disturbance throughout the 5-year period, except for the SAV IBI which did not perform well in 2011 or 2014.

**Fig. 6:**
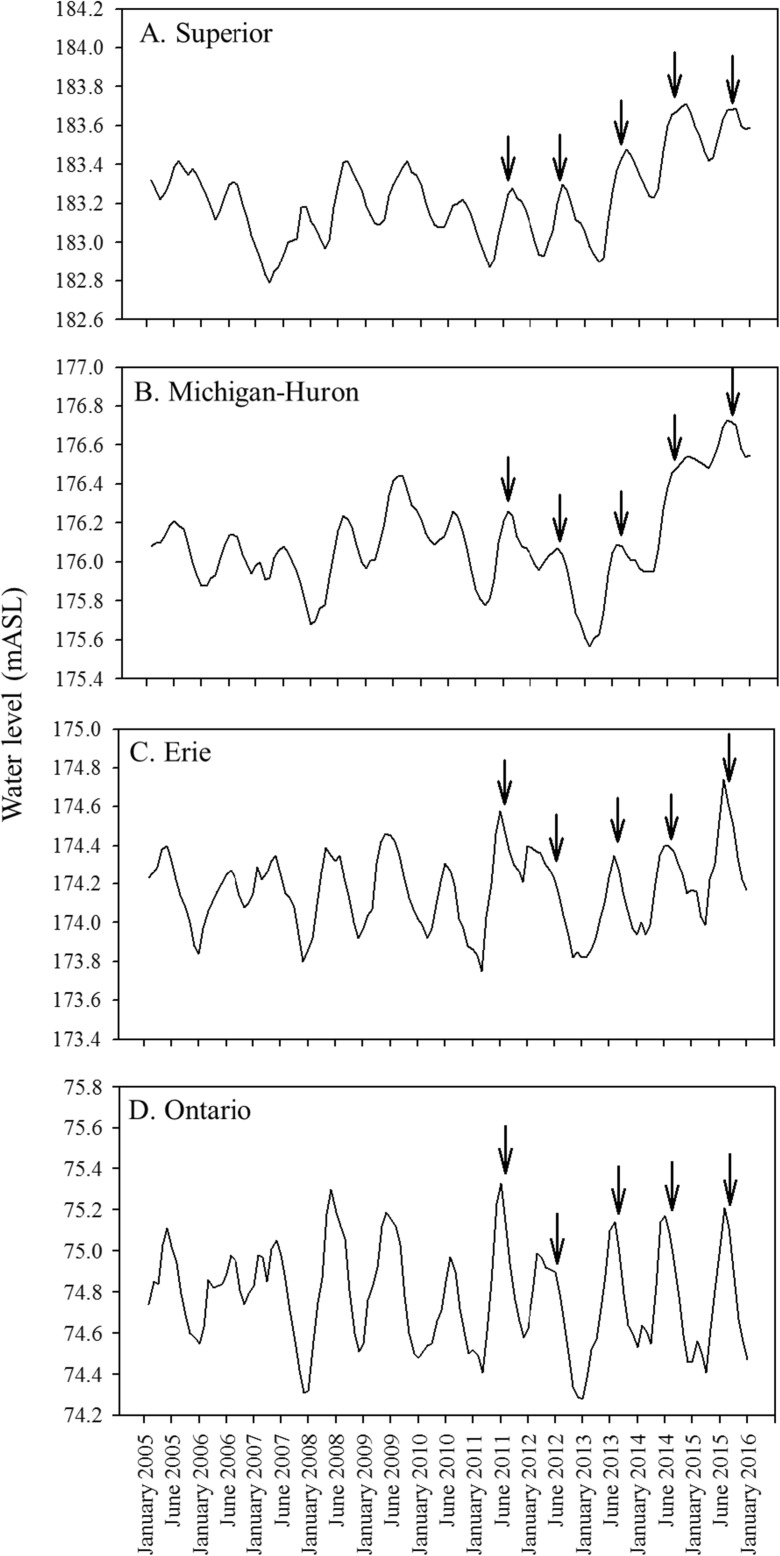
Great Lakes water levels (m above sea level, mASL) based on monthly mean levels, 2005–2015. Arrows represent approximate sampling periods (summers, 2011–2015). Data downloaded from the NOAA Great Lakes Environmental Research Laboratory Water Level Dashboard (https://www.glerl.noaa.gov/data/dashboard/data/)

While a small subset of metrics was common to all four IBIs (i.e., *Nonnative species richness* and *% Richness of species particularly sensitive to environmental degradation*), most metrics pertained uniquely to specific vegetation types. The identification of metrics that uniquely reflected the condition of different vegetation zones makes sense given that vegetation zonation in coastal wetlands is governed by broad-scale drivers such as hydrology (Keddy and Reznicek 1986; Lishawa et al. 2010), nutrient availability (Lougheed et al. 2001; Croft and Chow-Fraser 2007), wave exposure (Albert et al. 2005; Johnston et al. 2007), and sediment characteristics (Albert et al. 2005). These drivers, along with physical habitat differences among the vegetation types themselves, all influence faunal community structure (Burton et al. 2004; Uzarski et al. 2005; Cvetkovic et al. 2010). Much of this variability among vegetation zones is accounted for in the IBIs by the vegetation-specific sampling design and selection of different metrics for each vegetation type. Furthermore, this sampling design allows the IBIs to be used under different lake-level regimes, which addresses previous concerns that fish IBIs would not be valid for Great Lakes coastal wetlands because fluctuating water levels impart too much variability in habitat and community structure (Wilcox et al. 2002). For lakes Superior, Michigan, and Huron, the 5-year sampling period included 3 years of relatively low lake levels (2011–2013) as well as a period of rapidly rising lake levels (2014–2015) when water levels increased by approximately 0.5 m (Fig. 6). Water levels in lakes Erie and Ontario also varied over the sampling period (Fig. 6), although fluctuations in Lake Ontario levels were moderated by regulation of the outflow by the Moses-Saunders Dam (Wilcox and Xie 2007). The consistent performance of the IBIs over the 5-year period, which included marked water-level variation, suggests that the IBI approach is robust to inter-annual water-level fluctuations. However, as water levels continue to fluctuate in the future, IBI metrics should continue to be periodically re-evaluated to ensure their continued accuracy. Year-to-year changes in water levels have been shown to affect vegetation, invertebrate, and fish communities in these habitats (Gathman et al. 2005; Gathman and Burton 2011; Cooper et al. 2014; Langer et al. 2018). Other potential sources of inter-annual variation in fish communities such as shifting seasonal temperatures or changes in wave energy and currents along Great Lakes shorelines may also necessitate periodic re-evaluation of IBI metrics.

Although we developed IBIs for the four most common vegetation types encountered, the strength of relationships between IBI scores and anthropogenic disturbance varied. The weak correlations between the SAV-zone IBI and disturbance indices indicates that refinement of SAV metrics with additional data is desirable. It may also reflect the fact that the umbrella term “SAV” encompasses a variety of growth forms, which create different habitat conditions for fish, thus imparting a greater variability in the fish community for this vegetation type compared to the other vegetation types sampled. Nevertheless, we included the SAV-zone IBI in composite scores because it correlated significantly with disturbance indices using both the development and testing datasets and for three of the 5 years when relationships were evaluated on a per-year basis.

Wetlands in Lake Erie and western Lake Ontario, which had the highest percentages of surrounding agriculture and developed land, along with the poorest water quality, had more wetlands in the “moderately degraded” and “degraded” categories, based on IBI scores, than other regions of the Great Lakes (Fig. 5). This finding is consistent with previous studies evaluating anthropogenic impacts to coastal habitats throughout the Great Lakes (e.g., Danz et al. 2007; Trebitz et al. 2007; Morrice et al. 2008). Wetlands in the southern portion of the Great Lakes basin are more degraded than those to the north because the physical setting of the lower lakes provides for better agricultural growing conditions, and consequently a larger human population has settled in the south (Huang and Méthot 2015). Lake Superior wetlands, which tend to have the least surrounding agriculture and developed land and highest water quality, had relatively high IBI scores in general. Wetlands in lakes Huron and Michigan tended to have moderate IBI scores, except for northern Lake Huron, which had scores more similar to Lake Superior, reflecting the higher water quality (Chow-Fraser 2006) and the considerably lower proportion of agriculture and developed land in that region.

Finer scale geographic patterns in IBI scores also appeared to reflect the level of anthropogenic disturbance to which wetlands were subjected. For example, degraded wetlands in Lake Superior were located close to the Duluth-Superior Harbor near the city of Duluth, Minnesota and immediately adjacent to either residential/commercial development or commercial ship docks. Degraded wetlands in Lake Michigan occurred near highly agricultural or urbanized areas. One of these was located at the mouth of the Galien River in southern Michigan, which has a highly agricultural watershed and chronically high turbidity and dissolved N. Another degraded Lake Michigan wetland was located in southern Green Bay, an area with high turbidity and nutrient concentrations from the outflow of the Fox River (Sager and Richman 1991; De Stasio and Richman 1998). Brazner and Beals (1997) found lower fish diversity in wetlands located in the more eutrophic southern Green Bay compared with the northern portion of the bay. Seilheimer and Chow-Fraser (2006) also noted a gradient in the structure of fish communities from southern Green Bay to northern Green Bay and reported lower Wetland Fish Index scores in the southern bay. Our fish IBI reflected this north-to-south gradient of declining water and habitat quality in Green Bay (Fig. 5).

The gradient of increasing IBI scores from west to east in Lake Ontario (Fig. 5) coincides with decreasing areal extent of agricultural and urban land-cover and improving coastal water quality along the same longitudinal gradient. The coastal region from Toronto around the west end of Lake Ontario to Buffalo-Niagara is one of the most densely populated areas in the Great Lakes basin (Huang and Méthot 2015). Agricultural land-cover is also remarkably high in this region, and a mean of 44% of the area within our 20-km buffers surrounding wetlands situated between Toronto and Buffalo-Niagara were devoted to agriculture compared to the basin-wide mean of 29%. Past work has shown that coastal and nearshore water quality in the western half of Lake Ontario is consistently more degraded (e.g., higher dissolved nutrient concentrations, dissolved solids, turbidity) than in the eastern half of the lake (Cvetkovic and Chow-Fraser 2011; Makarewicz et al. 2012). Previous work also has shown that coastal wetland fish communities in western Lake Ontario tend to be less diverse and comprised of more tolerant species than those of eastern Lake Ontario (Uzarski et al. 2005; Cvetkovic and Chow-Fraser 2011). Our IBI scores captured this west-to-east gradient of increasing biotic integrity, with a transition occurring at approximately the 79th meridian, which lies just east of the Toronto and Buffalo-Niagara metropolitan areas (Fig. 5).

Similar to Lake Ontario, IBI scores in Lake Erie also tended to increase from west to east, corresponding to a gradient of improving water quality from Lake Erie’s western to eastern basins (Conroy et al. 2008; Kane et al. 2014). Lake Erie wetlands with relatively high IBI scores (i.e., “mildly impacted” wetlands) occurred in the Rondeau and Long Point Provincial Parks in Canada, and Presque Isle in Pennsylvania, all of which are protected areas known for their high floral and faunal diversity (Herdendorf 1987; Tiner et al. 2014).

### Comparison to other fish-based indicators in Great Lakes coastal wetlands

Seilheimer and Chow-Fraser (2006) developed the Wetland Fish Index (WFI) using fish and water quality data from 40 Great Lakes coastal wetlands. The WFI was based on a partial canonical correspondence analysis to ordinate fish species along environmental axes (primarily water quality features) to estimate species-environment associations and niche breadth. Although the WFI differs from our approach, a number of our metrics are consistent with the species-environment coefficients derived by Seilheimer and Chow-Fraser (2006). For example, common carp (*Cyprinus carpio*) and goldfish (*Carassius auratus*) both received very low coefficients in the WFI, indicating their tolerance of poor water quality. Similarly, the presence of these species reduced the scores for each of our IBIs because they are both nonnative, which influenced at least one metric in each of our IBIs, and because common carp was included specifically as a metric in three of our four IBIs (Table 2). We identified rock bass (*Ambloplites rupestris*) as an indicator of high quality habitat in three of our four IBIs; this species was similarly associated with good water quality in Seilheimer and Chow-Fraser (2006) and was found to have potential as a basin-wide indicator of coastal wetland health by Brazner et al. (2007). Seilheimer and Chow-Fraser (2006) found that a number of native cyprinids were associated with moderate-to-good water quality in Great Lakes coastal wetlands [e.g., blackchin shiner (*Notropis heterodon*), mimic shiner (*N. volucellus*), blacknose shiner (*N. heterolepis*), bluntnose minnow (*Pimephales notatus*), emerald shiner (*N. atherinoides*), and golden shiner (*Notemigonus crysoleucas*)]. These native cyprinids contributed to metrics in our IBIs as well. For example, *% Native Cyprinidae* and *Native Cyprinidae CPUE* were found to be useful metrics in cattail, bulrush, and SAV-zone IBIs. Other authors also have found metrics related to native cyprinid species to be useful indicators of habitat quality in lakes (Minns et al. 1994; Randall and Minns 2002), wetlands (Wilcox et al. 2002), and streams (Karr 1981; Fausch et al. 1984; Raburu and Masese 2012), likely due to the group’s generally high sensitivity to turbidity and other forms of environmental degradation (Whittier and Hughes 1998; Trebitz et al. 2007).

We found no positive relationship between species richness and habitat quality for any of the vegetation types, which is consistent with Seilheimer and Chow-Fraser (2006). They found a weak negative relationship between water quality and fish species richness. This result is likely because tolerant and often nonnative species tend to colonize degraded habitats and increase local species richness. Not surprisingly, therefore, we identified useful richness-based metrics based on whether species were native, and all four IBIs included at least one such metric. Others also have found that accounting for native vs. nonnative species improved richness-based metric performance in fish IBIs (Minns et al. 1994; Thoma 1999; Wilcox et al. 2002; Lyons 2012).

The fish-based IBIs developed by Uzarski et al. (2005) and evaluated further by Bhagat et al. (2007) included metrics for bulrush and cattail zones in Great Lakes coastal wetlands. We identified many of the same metrics as these previous efforts, with notable modifications and additions. In our bulrush zone IBI, *Evenness* was selected as a diversity metric instead of *Total taxon richness*. Neither *Total catch net-night*^*−1*^ nor *% Insectivores* (Uzarski et al. 2005) was retained. We identified *% Carnivore* as a metric, similar to the *% Carnivore richness* metric of Uzarski et al. (2005); interestingly however, the relationship between this metric and habitat quality was negative and nonlinear. Domination of an assemblage by just one or two carnivore taxa frequently resulted in > 90% carnivore relative abundance, which generally occurred in the most disturbed wetlands. Therefore, the scoring scheme for this metric was designed to detect fish assemblages that were drastically out of trophic balance compared to assemblages with a broader trophic base.

Our cattail zone IBI also differed from that of Uzarski et al. (2005) in a number of ways. We did not retain *Insectivorous Cyprinidae richness* but included *% Native Cyprinidae*. The cattail zone IBI of Uzarski et al. (2005) included six metrics related to Centrarchidae. We retained one of these, *Rock bass CPUE,* but the others were redundant and only marginally indicative of habitat quality in our analysis. Centrarchids did, however, contribute substantially to *% Richness nest spawners*, which was a useful indicators of habitat quality for cattail zones in our study. We also incorporated metrics related to species longevity, body size, and species sensitivity to anthropogenic disturbance, none of which were considered by Uzarski et al. (2005). Despite these modifications, we consider our current set of indices to be expanded and updated versions of the preliminary IBIs developed by Uzarski et al. (2005).

### Synopsis and applications

Anthropogenic stressors such as nutrient and sediment loading, which are generally related to human activities on the landscape, are important drivers of fish community structure in Great Lakes coastal wetlands (Brazner and Beals 1997; Seilheimer and Chow-Fraser 2006; Trebitz et al. 2009). Macrophytes also affect fish community structure in these habitats (Brazner and Beals 1997; Cvetkovic et al. 2010). Thus, we used an approach that leveraged the information contained in fish–habitat quality relationships while also accounting for the effects of macrophyte structure by sampling within discrete vegetation zones.

We developed the fish-based IBIs using a combination of water quality and land cover data for 470 coastal wetlands spanning the Great Lakes basin. By including locally collected water quality data, we incorporated wetland-scale variation in habitat quality that cannot be detected when using only landscape-scale data during IBI development. Additionally, the development of separate IBIs for different vegetation types is of particular importance in Great Lakes coastal wetlands because the number of vegetation types (thus, the degree of habitat variation) differs from wetland to wetland, and can differ within a given wetland from year to year if water levels fluctuate dramatically. The fact that different metrics were found to be useful in different vegetation types supports the idea that different fish species prefer different vegetation structure. Thus, we feel that the multiple vegetation type approach to IBI development is appropriate for representing the fish communities of Great Lakes coastal wetlands. Management agencies who are interested in using these methods but find the multi-zone sampling approach to be overly demanding on their time and resources may find it reasonable to reduce their effort by sampling in, and calculating IBIs for, only selected vegetation types.

Potential applications for the IBIs developed here include ranking fish community condition among wetlands to assist managers in identifying and prioritizing wetlands in need of restoration, tracking the efficacy of restoration investments throughout the Great Lakes, and assessing fish-related beneficial use impairments (BUIs) within Great Lakes Areas of Concern (see Annex 1 of the 2012 Great Lakes Water Quality Agreement). The IBIs also can be used by government agencies in Canada and the USA to report on State of the Lakes Ecosystem Conference (SOLEC) indicators and meet other obligations under the Great Lakes Water Quality Agreement (e.g., Annex 2, *Lakewide Management*; Annex 7, *Habitat and Species*; and Annex 10, *Science*). Each of these annexes contain requirements to monitor ecosystem health, and our fish-based IBIs are potential tools to accomplish this monitoring. Given that approximately 50% of Great Lakes coastal wetlands have been lost, and remaining wetlands suffer from a variety of human impacts, growing emphasis has been placed on protecting and restoring these ecosystems. Furthermore, as the last barrier to watershed runoff that enters the open waters of the Great Lakes, coastal wetlands occupy a critical position in protecting water quality and harboring unique biodiversity. Our approach provided indices that offer a robust means for classifying the condition of fish communities in wetlands, which can guide researchers and managers in identifying locations most in need of protection and restoration and for tracking conditions through time.

## Appendix

**Table 3:** Candidate fish assemblage-based metrics for IBI development (catch per unit effort, CPUE; smallmouth bass, SMB; largemouth bass, LMB). See Appendix Table 4 for taxonomic names of fishes

**1 – Diversity**	
Total species richness	Omnivore species richness
Shannon diversity	%Omnivore species richness
Evenness	Omnivore CPUE
	%Omnivore
**2 – Native vs. non-native**	Invertivore species richness
	%Invertivore species richness
Native species richness	Invertivore CPUE
%Native species richness	%Invertivore
Native species CPUE	
%Native	Benthic invertivore species richness
	%Benthic invertivore species richness
Non-native species richness	Benthic invertivore CPUE
%Non-native species richness	%Benthic invertivore
Non-native species CPUE	
%Non-native	Invertivorous Cyprinidae species richness
	%Invertivorous Cyprinidae species richness
**3a – Taxonomic community structure**	Invertivorous Cyprinidae CPUE
	%Invertivorous Cyprinidae
Centrarchidae species richness	
%Centrarchidae species richness	
Centrarchidae CPUE	**5 – Habitat affinity**
%Centrarchidae	
	Adult benthic habitat species richness
Centrarchidae species richness, excl. SMB & LMB	%Adult benthic habitat species
%Centrarchidae species richness, excl. SMB & LMB	Adult benthic habitat CPUE
Centrarchidae CPUE, excl. SMB & LMB	%Adult benthic habitat
%Centrarchidae, excl. SMB & LMB	
	Adult vegetation habitat species richness
Cyprinidae species richness	
%Cyprinidae species richness	%Adult vegetation habitat species
Cyprinidae CPUE	
%Cyprinidae	Adult vegetation habitat CPUE
Native Cyprinidae species richness	%Adult vegetation habitat
%Native Cyprinidae species richness	
Native Cyprinidae CPUE	
%Native Cyprinidae	Nest spawner richness
	%Nest spawner richness
Notropis species richness	
%Notropis species richness	Nest spawner CPUE
Notropis CPUE	%Nest spawners
%Notropis	
	Vegetation spawner richness
	%Vegetation spawner richness
**3b – 25 most common species (CPUE and Rel. Ab.)**	Vegetation spawner CPUE
White sucker	%Vegetation spawners
Black bullhead	
Rock bass	**6 – Species attributes**
Alewife	
Smallmouth bass	Long-lived species richness
Longnose gar	%Long-lived species richness
Largemouth bass	Long-lived species CPUE
Bluegill	%Long-lived species
*Lepomis*	
Black+brown bullhead	Short-lived species richness
Banded killifish	%Short-lived species richness
Blacknose shiner	Short-lived species CPUE
Bluntnose minnow	%Short-lived species
Bowfin	
Emerald shiner	Large and and XLarge species richness
Golden shiner	%Large and XLarge species richness
Johnny darter	Large and XLarge species CPUE
Pumpkinseed	%Large and Xlarge species
Round goby	
Sand shiner	Small species richness
Spottail shiner	%Small species richness
Yellow perch	Small species CPUE
Blackchin shiner	%Small species
Brook silversides	
Brook stickleback	High and XHigh temperature spawner species richness
Common carp	%High and XHigh temperature spawner species richness
Common shiner	High and XHigh temperature species CPUE
	%High and XHigh temperature spawner species
**4 – Feeding ecology**	
	**7 – Sensitivity/tolerance to human disturbance**
Piscivore species richness	
%Piscivore species richness	Number of species particularly sensitive to environmental degradation
Piscivore CPUE	%Species richness particularly sensitive to environmental degradation
%Piscivore	Species particularly sensitive to environmental degradation CPUE
	%Particularly sensitive to degradation
Carnivore species richness	
%Carnivore species richness	Number of species particularly tolerant to environmental degradation
Carnivore CPUE	%Species particularly tolerant to environmental degradation
%Carnivore	Species particularly tolerant to environmental degradation CPUE
	%Particularly tolerant to degradation

**Table 4:** Classification of fish taxa. Fish taxa were classified into adult habitat (HAB), adult food (FOOD), lifespan (LIFE), TROPHIC, total length (LENGTH), spawning habitat (SPAWN_H), spawning temperature (SPAWN_T), overall tolerance (TOL), and endemism (ENDEM) groups. Sources for HAB, LONG, TROPHIC, LENGTH, SPAWN_H, and SPAWN_T information include Goldstein and Simon (1999), Halliwell et al. (1999), Hubbs et al. (2004), Pflieger (1975), Simon (1999), and Whittier and Hughes (1998). Sources for FOOD and TOL include Plafkin et al. (1989) and Lyons (2006, 2012)

	Trait	HAB	FOOD	LIFE	TROPHIC	LENGTH
	Trait codes	bt, benthic	fh, fish	hg, >15 yrs.	ff, filter feeder	xl, >600 mm
		md, mud	mi, macroinvertebrates	md, 5–15 yrs.	gf, generalist	lg, 301–600 mm
		pl, pool	ov, omnivore	lw, <5 yrs.	in, insectivore	md, 101–300 mm
		pz, pelagic	pt, plants		iv, invertivore	sm, 0–100 mm
		rf, riffle	pz, phytoplankton.		ov, omnivore	
		rk, rock			pv, piscivore	
		su, surface				
Species name	Common name	vg, vegetation				
*Alosa pseudoharengus*	Alewife	pz	pz	lw	ff	md
*Ambloplites rupestris*	Rock bass	vg	ov	md	pv	md
*Ameiurus melas*	Black bullhead	bt	ov	md	in	md
*Ameiurus melas* or *A. nebulosus*	Black or brown bullhead	bt	ov	lw	in	md
*Ameiurus natalis*	Yellow bullhead	bt	ov	md	in	md
*Ameiurus nebulosus*	Brown bullhead	bt	ov	lw	in	md
*Amia calva*	Bowfin	hy	fh	md	pv	lg
*Anguilla rostrate*	American eel	bt	ov	md	pv	xl
*Aphredoderus sayanus*	Pirate perch	md	ov	lw	in	sm
*Aplodinotus grunniens*	Freshwater drum	bt	mi	hg	iv	lg
*Carassius auratus*	Goldfish	bt	ov	md	ov	md
*Carpiodes cyprinus*	Quillback	bt	ov	md	ov	lg
Catostomidae	Suckers	bt	ov	md	ov	md
*Catostomus commersonii*	White sucker	bt	ov	md	ov	md
Centrarchidae	Sunfishes	vg	ov	md	in	md
*Lepomis gulosus*	Warmouth	vg	ov	md	pv	md
*Coregonus artedi*	Lake cisco	pz	pz	md	ff	md
*Cottus bairdii*	Mottled sculpin	gn	ov	lw	in	sm
*Cottus cognatus*	Eastern slimy sculpin	bt	ov	lw	in	sm
*Couesius plumbeus*	Lake chub	gn	ov	lw	in	md
*Culaea inconstans*	Brook stickleback	vg	ov	lw	in	sm
*Cyprinella spiloptera*	Spotfin shiner	bt	ov	lw	in	sm
Cyprinidae	Carps and minnows	bt	ov	md	ov	lg
*Cyprinus carpio*	Common carp	bt	ov	md	ov	lg
*Dorosoma cepedianum*	Gizzard shad	pz	pz	lw	ov	lg
*Esox americanus*	Grass pickerel	vg	fh	lw	pv	md
*Esox lucius*	Northern pike	vg	fh	md	pv	lg
*Esox masquinongy*	Muskellunge	vg	fh	hg	pv	xl
*Esox niger*	Chain pickerel	vg	fh	md	pv	lg
*Etheostoma*	Darters	bt	ov	lw	in	sm
*Etheostoma caeruleum*	Rainbow darter	bt	mi	lw	in	sm
*Etheostoma chlorosomum*	Bluntnose darter	bt	mi	lw	in	sm
*Etheostoma exile*	Iowa darter	bt	ov	lw	in	sm
*Etheostoma microperca*	Least darter	bt	mi	lw	in	sm
*Etheostoma nigrum*	Johnny darter	bt	mi	hg	in	sm
*Fundulus diaphanous*	Banded killifish	su	ov	lw	in	sm
*Gambusia affinis*	Western mosquitofish	su	mi	lw	in	sm
*Gasterosteus aculeatus*	Threespine stickleback	vg	ov	lw	in	sm
*Gymnocephalus cernuus*	Eurasian ruffe	pl	ov	md	ov	md
*Hybognathus hankinsoni*	Brassy minnow	vg	ov	lw	ov	sm
*Ictaluridae*	North American catfishes	bt	ov	md	pv	lg
*Ictalurus punctatus*	Channel catfish	bt	ov	md	pv	lg
*Ictiobus cyprinellus*	Bigmouth buffalo	md	ov	md	in	lg
*Labidesthes sicculus*	Brook silverside	pz	ov	md	in	sm
Lepisosteidae	Gars	su	fh	hg	pv	lg
*Lepisosteus oculatus*	Spotted gar	su	fh	hg	pv	xl
*Lepisosteus osseus*	Longnose gar	su	fh	hg	pv	lg
*Lepisosteus platostomus*	Shortnose Gar	su	fh	hg	pv	lg
*Lepomis*	Sunfishes	vg	mi	md	in	md
*Lepomis cyanellus*	Green sunfish	vg	fh	md	in	md
*Lepomis gibbosus*	Pumpkinseed	vg	ov	md	in	md
*Lepomis humilis*	Orangespotted sunfish	vg	ov	lw	in	sm
*Lepomis macrochirus*	Bluegill	vg	mi	md	in	md
*Lepomis megalotis*	Longear sunfish	vg	ov	md	in	sm
*Lota lota*	Burbot	bt	fh	md	pv	lg
*Luxilus chrysocephalus*	Striped shiner	vg	ov	md	in	sm
*Luxilus cornutus*	Common shiner	vg	ov	md	in	sm
*Lythrurus umbratilis*	Redfin shiner	vg	ov	lw	in	sm
*Margariscus margarita*	Pearl dace	pz	ov	lw	in	sm
*Micropterus dolomieu*	Smallmouth bass	rk	ov	md	pv	md
*Micropterus salmoides*	Largemouth bass	vg	ov	md	pv	md
*Minytrema melanops*	Spotted sucker	bt	ov	md	in	md
*Morone Americana*	White perch	pz	ov	md	pv	md
*Morone chrysops*	White bass	su	ov	lw	pv	md
*Moxostoma anisurum*	Silver redhorse	bt	ov	md	in	md
*Moxostoma breviceps*	Shorthead redhorse	bt	ov	md	in	md
*Moxostoma erythrurum*	Golden redhorse	bt	ov	md	in	md
*Moxostoma valenciennesi*	Greater redhorse	bt	ov	md	in	lg
*Neogobius melanostomus*	Round goby	bt	ov	lw	ov	md
*Nocomis biguttatus*	Horneyhead chub	bt	ov	lw	in	sm
*Notemigonus crysoleucas*	Golden shiner	vg	ov	md	ov	md
*Notropis*	Shiners	vg	ov	lw	in	sm
*Notropis anogenus*	Pugnose shiner	gn	pt	lw	in	sm
*Notropis atherinoides*	Emerald shiner	pz	mi	lw	in	sm
*Notropis bifrenatus*	Bridled shiner	vg	ov	lw	in	sm
*Notropis heterodon*	Blackchin shiner	su	ov	lw	in	sm
*Notropis heterolepis*	Blacknose shiner	vg	ov	lw	in	sm
*Notropis hudsonius*	Spottail shiner	gn	ov	lw	in	sm
*Notropis rubellus*	Rosyface shiner	rf	mi	lw	in	sm
*Notropis stramineus*	Sand shiner	vg	ov	lw	in	sm
*Notropis volucellus*	Mimic shiner	vg	ov	lw	in	sm
*Noturus gyrinus*	Tadpole madtom	bt	ov	lw	in	sm
*Noturus miurus*	Brindled madtom	bt	mi	lw	in	sm
*Oncorhynchus tshawytscha*	Chinook salmon	pz	ov	md	pv	xl
*Osmerus mordax*	Rainbow smelt	pz	ov	md	iv	md
*Perca flavescens*	Yellow perch	bt	ov	md	in	md
*Percina caprodes*	Logperch	bt	ov	lw	in	sm
*Percopsis omiscomaycus*	Trout-perch	bt	ov	lw	in	sm
*Petromyzon marinus*	Sea lamprey	pz	fh	lw	pv	lg
*Phoxinus eos*	Northern redbelly dace	bt	pt	lw	gf	sm
*Phoxinus neogaeus*	Finescale dace	bt	mi	md	in	sm
*Pimephales notatus*	Bluntnose minnow	su	ov	lw	ov	sm
*Pimephales promelas*	Fathead minnow	su	ov	lw	ov	sm
*Pomoxis*	Black or white crappie	vg	ov	md	pv	md
*Pomoxis annularis*	White crappie	pz	ov	md	pv	md
*Pomoxis nigromaculatus*	Black crappie	bt	mi	md	in	lg
*Proterorhinus marmoratus*	Tubenose goby	bt	ov	lw	in	sm
*Pungitius pungitius*	Ninespine stickleback	vg	ov	lw	in	sm
*Rhinichthys obtusus*	Western blacknose dace	rf	mi	lw	gf	sm
*Rhinichthys cataractae*	Longnose dace	rf	mi	lw	in	sm
*Sander*	Pikeperches	bt	fh	hg	pv	lg
*Sander vitreus*	Walleye	bt	fh	hg	pv	lg
*Scardinius erythrophthalmus*	Rudd	bt	mi	lw	ov	md
*Semotilus atromaculatus*	Creek chub	bt	ov	md	gf	md
*Umbra*	Mudminnows	vg	ov	lw	in	sm
*Umbra limi*	Central mudminnow	vg	ov	lw	in	sm
	Trait	SPAWN_H	SPAWN_T	TOL	ENDEM	
	Trait codes	bc, broadcast	xh, extra high (>21)	i, intolerant	n, nonnative	
		cv, under rocks	hg, high (16–20)	m, intermediate	y, native	
		lb, livebearer	md, moderate (11–15)	t, tolerant		
		ns, nests or redds	lw, low (4–10)			
		pl, pool				
		rk, riffles or shoals				
Species name	Common name	vg, vegetation				
*Alosa pseudoharengus*	Alewife	bc	md	m	n	
*Ambloplites rupestris*	Rock bass	ns	hg	m	y	
*Ameiurus melas*	Black bullhead	ns	xh	m	y	
*Ameiurus melas* or *A. nebulosus*	Black or brown bullhead	ns	xh	t	y	
*Ameiurus natalis*	Yellow bullhead	ns	xh	t	y	
*Ameiurus nebulosus*	Brown bullhead	ns	xh	t	y	
*Amia calva*	Bowfin	ns	hg	m	y	
*Anguilla rostrate*	American eel	bc	hg	m	y	
*Aphredoderus sayanus*	Pirate perch	ns	xh	m	y	
*Aplodinotus grunniens*	Freshwater drum	bc	xh	m	n	
*Carassius auratus*	Goldfish	vg	hg	t	n	
*Carpiodes cyprinus*	Quillback	bc	xh	m	y	
Catostomidae	Suckers	rk	md	t	y	
*Catostomus commersonii*	White sucker	rk	md	t	y	
Centrarchidae	Sunfishes	ns	hg	m	y	
*Lepomis gulosus*	Warmouth	ns	xh	m	y	
*Coregonus artedi*	Lake cisco	bc	lw	m	y	
*Cottus bairdii*	Mottled sculpin	cv	lw	i	y	
*Cottus cognatus*	Eastern slimy sculpin	cv	lw	m	y	
*Couesius plumbeus*	Lake chub	bc	hg	m	y	
*Culaea inconstans*	Brook stickleback	ns	hg	m	y	
*Cyprinella spiloptera*	Spotfin shiner	cv	xh	m	y	
Cyprinidae	Carps and minnows	vg	xh	m	u	
*Cyprinus carpio*	Common carp	vg	xh	t	n	
*Dorosoma cepedianum*	Gizzard shad	bc	md	m	y	
*Esox americanus*	Grass pickerel	vg	lw	m	y	
*Esox lucius*	Northern pike	vg	lw	m	y	
*Esox masquinongy*	Muskellunge	vg	lw	m	y	
*Esox niger*	Chain pickerel	vg	lw	m	y	
*Etheostoma*	Darters	vg	md	m	y	
*Etheostoma caeruleum*	Rainbow darter	ns	md	m	y	
*Etheostoma chlorosomum*	Bluntnose darter	vg	md	m	y	
*Etheostoma exile*	Iowa darter	vg	md	m	y	
*Etheostoma microperca*	Least darter	vg	md	m	y	
*Etheostoma nigrum*	Johnny darter	cv	md	m	y	
*Fundulus diaphanous*	Banded killifish	vg	xh	t	y	
*Gambusia affinis*	Western mosquitofish	lb	xh	m	n	
*Gasterosteus aculeatus*	Threespine stickleback	ns	hg	m	y	
*Gymnocephalus cernuus*	Eurasian ruffe	vg	md	m	n	
*Hybognathus hankinsoni*	Brassy minnow	vg	hg	m	y	
Ictaluridae	North American catfishes	cv	xh	m	y	
*Ictalurus punctatus*	Channel catfish	cv	xh	m	y	
*Ictiobus cyprinellus*	Bigmouth buffalo	vg	hg	m	y	
*Labidesthes sicculus*	Brook silverside	bc	xh	m	y	
Lepisosteidae	Gars	vg	hg	m	y	
*Lepisosteus oculatus*	Spotted gar	vg	hg	m	y	
*Lepisosteus osseus*	Longnose gar	vg	hg	m	y	
*Lepisosteus platostomus*	Shortnose Gar	vg	hg	m	y	
*Lepomis*	Bluegill or pumpkinseed	ns	hg	m	y	
*Lepomis cyanellus*	Green sunfish	ns	hg	t	y	
*Lepomis gibbosus*	Pumpkinseed	ns	hg	m	y	
*Lepomis humilis*	Orangespotted sunfish	ns	hg	m	n	
*Lepomis macrochirus*	Bluegill	ns	hg	m	y	
*Lepomis megalotis*	Longear sunfish	ns	hg	i	y	
*Lota lota*	Burbot	bc	lw	m	y	
*Luxilus chrysocephalus*	Striped shiner	rk	xh	m	y	
*Luxilus cornutus*	Common shiner	rk	xh	m	y	
*Lythrurus umbratilis*	Redfin shiner	ns	xh	m	y	
*Margariscus margarita*	Pearl dace	rk	md	m	y	
*Micropterus dolomieu*	Smallmouth bass	ns	hg	m	y	
*Micropterus salmoides*	Largemouth bass	ns	hg	m	y	
*Minytrema melanops*	Spotted sucker	rk	hg	m	y	
*Morone Americana*	White perch	bc	hg	m	n	
*Morone chrysops*	White bass	bc	hg	m	y	
*Moxostoma anisurum*	Silver redhorse	rk	hg	m	y	
*Moxostoma breviceps*	Shorthead redhorse	bc	hg	m	y	
*Moxostoma erythrurum*	Golden redhorse	rk	hg	m	y	
*Moxostoma valenciennesi*	Greater redhorse	rk	hg	i	y	
*Neogobius melanostomus*	Round goby	rk	md	t	n	
*Nocomis biguttatus*	Horneyhead chub	ns	hg	i	y	
*Notemigonus crysoleucas*	Golden shiner	vg	hg	t	y	
*Notropis*	Shiners	vg	xh	m	y	
*Notropis anogenus*	Pugnose shiner	vg	xh	i	y	
*Notropis atherinoides*	Emerald shiner	rk	xh	m	y	
*Notropis bifrenatus*	Bridled shiner	vg	xh	i	y	
*Notropis heterodon*	Blackchin shiner	vg	xh	i	y	
*Notropis heterolepis*	Blacknose shiner	vg	xh	i	y	
*Notropis hudsonius*	Spottail shiner	rk	xh	m	y	
*Notropis rubellus*	Rosyface shiner	bc	xh	i	y	
*Notropis stramineus*	Sand shiner	vg	xh	m	y	
*Notropis volucellus*	Mimic shiner	vg	xh	i	y	
*Noturus gyrinus*	Tadpole madtom	cv	xh	m	y	
*Noturus miurus*	Brindled madtom	cv	xh	i	y	
*Oncorhynchus tshawytscha*	Chinook salmon	ns	lw	m	n	
*Osmerus mordax*	Rainbow smelt	bc	lw	m	n	
*Perca flavescens*	Yellow perch	vg	hg	m	y	
*Percina caprodes*	Logperch	ns	hg	m	y	
*Percopsis omiscomaycus*	Trout-perch	bc	hg	m	y	
*Petromyzon marinus*	Sea lamprey	ns	lw	m	n	
*Phoxinus eos*	Northern redbelly dace	vg	hg	m	y	
*Phoxinus neogaeus*	Finescale dace	cv	hg	m	y	
*Pimephales notatus*	Bluntnose minnow	ns	xh	t	y	
*Pimephales promelas*	Fathead minnow	cv	xh	t	y	
*Pomoxis*	Black or white crappie	ns	hg	m	y	
*Pomoxis annularis*	White crappie	ns	hg	m	y	
*Pomoxis nigromaculatus*	Black crappie	bc	lw	m	y	
*Proterorhinus marmoratus*	Tubenose goby	rk	md	t	n	
*Pungitius pungitius*	Ninespine stickleback	ns	md	m	y	
*Rhinichthys obtusus*	Western blacknose dace	ns	hg	t	y	
*Rhinichthys cataractae*	Longnose dace	rk	hg	i	y	
*Sander*	Pikeperches	rk	lw	m	y	
*Sander vitreus*	Walleye	rk	lw	m	y	
*Scardinius erythrophthalmus*	Rudd	vg	hg	t	n	
*Semotilus atromaculatus*	Creek chub	ns	md	t	y	
*Umbra*	Mudminnows	vg	md	t	y	
*Umbra limi*	Central mudminnow	vg	md	t	y	
